# Outcomes of neoadjuvant chemotherapy and radical hysterectomy for locally advanced cervical cancer at Kigali University Teaching Hospital, Rwanda: a retrospective descriptive study

**DOI:** 10.1186/s12905-024-03024-z

**Published:** 2024-03-30

**Authors:** Eugene Ngabonziza, Rahel Ghebre, Rebecca J. DeBoer, Diomede Ntasumbumuyange, Urania Magriples, Jessica George, Surbhi Grover, Lisa Bazzett-Matabele

**Affiliations:** 1 University of Rwanda, Kigali, Rwanda; 2 University of Minnesota Medical School, Minneapolis, MN USA; 3 University of California, San Francisco, CA USA; 4 Yale School of Medicine, New Haven, CT USA; 5 University of California, Irvine, CA USA; 6 University of Pennsylvania, Philadelphia, PA USA; 7 Department of OBGYN, University of Botswana, Sir Ketumile Masire Teaching Hospital, Pvt Bag, 00713 Gaborone, Botswana

**Keywords:** Cervical cancer, Neoadjuvant chemotherapy, Radical hysterectomy, Limited resources

## Abstract

**Background:**

Half of countries in Africa lack access to radiation (RT), which is essential for standard treatment of locally advanced cervical cancers. We evaluated outcomes for patients treated with neoadjuvant chemotherapy (NACT) followed by radical hysterectomy in settings where no RT is available.

**Methods:**

We performed a retrospective descriptive study of all patients with FIGO stage IB2-IIA2 and some exceptional stage IIB cases who received NACT and surgery at Kigali University Teaching Hospital in Rwanda. Patients were treated with NACT consisting of carboplatin and paclitaxel once every 3 weeks for 3–4 cycles before radical hysterectomy. We calculated recurrence rates and overall survival (OS) rate was determined by Kaplan-Meier estimates.

**Results:**

Between May 2016 and October 2018, 57 patients underwent NACT and 43 (75.4%) were candidates for radical hysterectomy after clinical response assessment. Among the 43 patients who received NACT and surgery, the median age was 56 years, 14% were HIV positive, and FIGO stage distribution was: IB2 (32.6%), IIA1 (7.0%), IIA2 (51.2%) and IIB (9.3%). Thirty-nine (96%) patients received 3 cycles and 4 (4%) received 4 cycles of NACT. Thirty-eight (88.4%) patients underwent radical hysterectomy as planned and 5 (11.6%) had surgery aborted due to grossly metastatic disease. Two patients were lost to follow up after surgery and excluded from survival analysis. For the remaining 41 patients with median follow-up time of 34.4 months, 32 (78%) were alive with no evidence of recurrence, and 8 (20%) were alive with recurrence. One patient died of an unrelated cancer. The 3-year OS rate for the 41 patients who underwent NACT and surgery was 80.8% with a recurrence rate of 20%.

**Conclusions:**

Neoadjuvant chemotherapy with radical hysterectomy is a feasible treatment option for locally advanced cervical cancer in settings with limited access to RT. With an increase in gynecologic oncologists skilled at radical surgery, this approach may be a more widely available alternative treatment option in countries without radiation facilities.

## Background

In 2020, there were an estimated 604,000 new cases and 342,000 deaths due to cervical cancer worldwide, with 84% of new cases and 88% of deaths occurring in low- and middle-income countries (LMICs) [1]. In addition, cervical cancer numbers are expected to continue to rise to 700,000 cases and 400,000 deaths by 2030, with LMICs continuing to suffer the greatest burden [2]. Despite continued efforts and advancements in prevention and treatment, cervical cancer remains the leading cause of cancer-related death in women in sub-Saharan Africa (SSA) [3] as most women present with advanced stage disease [4] either due to limited screening opportunities or lack of access to definitive treatment once diagnosed. Most efforts in reducing cervical cancer burden in Africa have focused on screening and primary prevention, with a lack of studies on invasive cervical cancer treatment and survival outcomes relevant to limited resource settings [5].

Since 1999, concurrent platinum-based chemotherapy and radiotherapy (CRT) has been the standard treatment globally for locally advanced cervical cancer, where available, including stages IB2-IVA [International Federation of Gynaecology and Obstetrics (FIGO) 2009 staging] or IB3-IVA (FIGO 2018 staging). Access to radiation facilities remains a challenge to treatment in limited-resource countries. Neoadjuvant Chemotherapy (NACT) followed by radical surgery (NACT-S) for locally advanced cervical cancer compared to CRT has not been shown to improve cervical cancer survival [6], although it does offer an option for surgical management of locally advanced cervical cancer and is thus considered a reasonable alternative in settings with limited access to radiation therapy (RT). Therefore, NACT-S has been recommended as an alternative treatment for FIGO stages IB2- IIB cervical cancer in resource stratified consensus guidelines when radiation is unavailable [7, 8]. However, data on outcomes of patients with locally advanced disease treated with NACT-S in SSA is lacking.

In Rwanda, prior to 2014, patients with very early-stage cervical cancer (IA1) were offered simple hysterectomy by a general gynecologist, and patients with more advanced disease were offered RT at Uganda Cancer Institute (UCI), as Rwanda had no gynecologic oncology surgeons or RT facilities [9]. In 2014, gynecologic oncologists began working in Rwanda through the Human Resources for Health program [10], and advanced early stage (IA2-IB1, IIA1 felt to be resectable based on size and location) patients were then treated surgically with radical hysterectomy rather than CRT. In early 2016, the radiotherapy machine at UCI broke down beyond repair, creating an urgent need for alternative treatments for locally advanced cervical cancer. RT was made available to Rwandan patients a few months later at Nairobi Hospital in Kenya through financial support from the organization Partners in Health, but for a much smaller number of patients due to increased distance and cost [11]. A treatment protocol was then initiated for locally advanced cervical cancer consisting of NACT-S. For patients with early locally advanced stage (IB2-IIB) cervical cancer, this approach is a feasible alternative in settings with limited or no radiation facilities as it can reduce micro metastasis and improve operability [12]. Although this treatment strategy is being used in LMICs, follow-up and survival rates for most of these patients go unreported due to the lack of capacity and infrastructure to track patients and collect good quality clinical and outcomes data [13]. Therefore, the purpose of our study was to determine the clinical and pathological response rates, recurrence rates, and overall survival (OS) for patients with FIGO 2009 stages IB2-IIB cervical cancer who were treated with NACT and radical surgery in Rwanda when RT was unavailable.

## Methods

### Study design and data collection

Retrospective data were collected on all cervical cancer patients who were treated with NACT with a plan for type III radical hysterectomy (removal of the uterus, cervix, upper vagina and parametrium) and pelvic lymphadenectomy from May 2016 to October 2018. Patients were identified using hospital registers and admission files. Data pertaining to demographics, histopathologic diagnosis, number of NACT cycles received, clinical and pathological response to NACT, surgical complications, surgical pathology results, disease recurrence, and survival were recorded.

### Treatment protocol

Patients were staged according to FIGO 2009 staging. Due to limited resources and access to radiographs, patients were staged and triaged using clinical exam and bedside abdominal/pelvic ultrasound. Ultrasound was used to rule out hydronephrosis and liver metastases. Patients with clinical stage IB2, IIA1 tumors felt unlikely to result in clear margins due to size and/or location, IIA2 tumors, and a few rare exceptions of stage IIB tumors, were considered for NACT-S. As per our protocol, ideally, patients with stages IIB and above would have been treated with CRT, but during periods of limited radiation access, exceptions were made to include stage IIB in this NACT protocol. Cervical cancer diagnosis was confirmed with histology prior to treatment with NACT.

All cervical cancer patients were examined by a gynecologic oncologist at the University Teaching Hospital of Kigali (CHUK) and those who were candidates for NACT based on clinical staging were sent to Butaro Cancer Center (BCC) to receive carboplatin AUC 6 and paclitaxel 175 mg/m^2^ every 21 days for 3–4 cycles based on established NACT protocol [14]. Those with early-stage cervical cancer (IA1/2 or IB1) deemed eligible for curative surgical management, or with more advanced disease (stage III-IV) deemed eligible for CRT or palliative therapy, as well as those with concomitant cancer in other organs, were excluded from the treatment protocol.

After receiving NACT, patients returned to CHUK for re-evaluation by a gynecologic oncologist to assess response to treatment and determine which patients were candidates for radical surgery. Cervical tumor size based on clinical exam pre- and post-NACT was documented. Patients with partial or complete response to chemotherapy were defined as surgically resectable and were scheduled for radical hysterectomy and pelvic lymphadenectomy. Preoperative investigations included complete blood count, HIV test, liver and renal functions test, and routine chest x-ray and EKG for women aged 60 years and above. Patients with no or minimal response to NACT were referred for CRT.

### Patient follow-up

After radical hysterectomy, patients were followed up 4–6 weeks postoperatively to review their surgical pathology results. From these results, it was determined if surgery alone was adequate for cure or if adjuvant CRT was indicated for high-risk features including residual tumor size > 4 cm, positive surgical margins, or pelvic or paraaortic lymph nodes metastases. Depth of stromal invasion and lymphovascular space invasion were unavailable on histopathology reports at that time. For patients adequately treated with surgery alone post-NACT, routine surveillance with pelvic examination every 3 months for the first 2 years then every 6 months for the subsequent 3 years was recommended [8]. Patients found to have metastatic disease intra-operatively or by pathology post-operatively were referred for definitive CRT.

### Statistical analysis

Data collection and entry was done using Epidata 3.1. Descriptive statistics were used to summarize patient and clinical characteristics for the cohort of patients who received NACT-S. The survival rate was estimated via the Kaplan-Meier method. Univariable Cox hazards regression model was used to evaluate factors associated with overall survival in all patients. Statistical analysis was performed using RStudio 2020 (RStudio Team, Boston, MA) and statistical significance was set at a threshold of *p* < 0.05.

### Ethics

Institutional Review Board approval was obtained from the University of Rwanda College of Medicine Health Sciences and Kigali University Teaching Hospital.

## Results

**Fig. 1:**
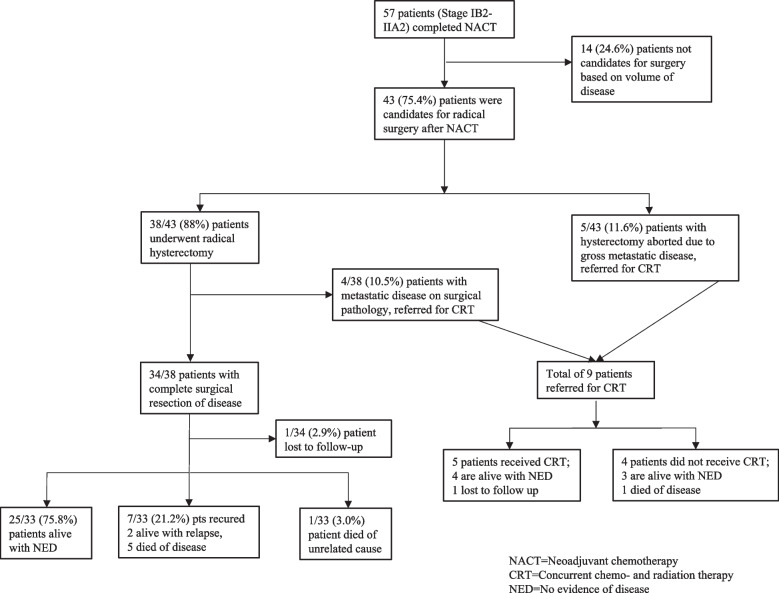
Patient flow diagram

Between May 2016 and October 2018, 57 women with stages IB2-IIB cervical cancer underwent NACT and 43 (75.4%) were identified as candidates for radical hysterectomy after clinical response assessment by a gynecologic oncologist. The remaining 14 (25%) patients had inadequate clinical response to NACT and were referred for CRT. A patient flow diagram is presented in Fig. 1. Among the 43 patients who received NACT and surgery, the median age was 56.1 years (range 36–78 years). Six (14%) of patients were HIV-positive. Histopathologic diagnosis was squamous cell carcinoma in 40 (93%) and adenocarcinoma in 3 (7%). FIGO stages were IB2–14 patients (32.6%), IIA1-3 patients (7.0%), IIA2–22 patients (51.2%) and IIB-4 patients (9.3%). Thirty-nine (96%) patients received 3 and 4 (4%) received 4 cycles of NACT. Cervical tumor size was evaluated by clinical exam before and after NACT with a mean and median reduction of 3.9 cm (standard deviation [SD] 1.17) and 4.0 cm (interquartile range [IQR] 3.00–5.00), respectively (range 2-7 cm). Preoperative haemoglobin was ≥12 g/dl in 27 (62.8%) patients and preoperative platelet count was ≥150,000 in 39 (90.7%). Patient demographics and clinical assessment before radical hysterectomy are presented in Table 1.

**Table 1:** Demographics

Characteristic	Overall Cohort
Overall cases, *n* (%)	43 (100)
Age (years)	
Mean (SD)	56.1 (10.8)
Median (IQR)	56.0 (50.0–63.0)
Age (years), *n* (%)	
35–45	6 (14.6)
46–55	14 (34.1)
56–65	16 (37.2)
> 65	7 (17.1)
HIV status, *n* (%)	
Negative	37 (86.0)
Positive	6 (14.0)
Clinical stage, *n* (%)	
IB2	14 (32.6)
IIA1	3 (7.0)
IIA2	22 (51.2)
IIB	4 (9.3)
Hemoglobin (g/dL)	
Mean (SD)	12.2 (1.4)
Median (IQR)	12.5 (11.6–13.2)
Hemoglobin (g/dL), *n* (%)	
< 12	16 (37.2)
≥ 12	27 (62.8)
Platelets (K)	
Mean (SD)	243.0 (78.7)
Median (IQR)	211.0 (187.5–286.0)
Platelets (K), *n* (%)	
< 150	4 (9.3)
≥ 150	39 (90.7)
Reduction in Tumor Size with NACT (cm)	
Mean (SD)	3.9 (1.2)
Median (IQR)	4.0 (3.0–5.0)
Histopathology before NACT, *n* (%)	
Squamous carcinoma	40 (93.0)
Adenocarcinoma	3 (7.0)
Number of cycles of NACT (cycles), *n* (%)	
3	39 (90.7)
4	4 (9.3)

*SD* standard deviation, *IQR* interquartile range, *HIV* human immunodeficiency virus

Thirty-eight of the 43 (88.4%) surgical patients underwent radical hysterectomy after NACT as planned. Five of 43 (11.6%) had surgery aborted due to metastatic disease found intra-operatively by clinical impression as no frozen section was available. Patient treatment summary and outcomes are presented in Table 2. Perioperative complications included ureteral injury (*n* = 1), bleeding requiring transfusion (*n* = 1), pelvic abscess (*n* = 1), urinary tract infection (*n* = 2), and superficial surgical site infection (*n* = 4).

**Table 2:**
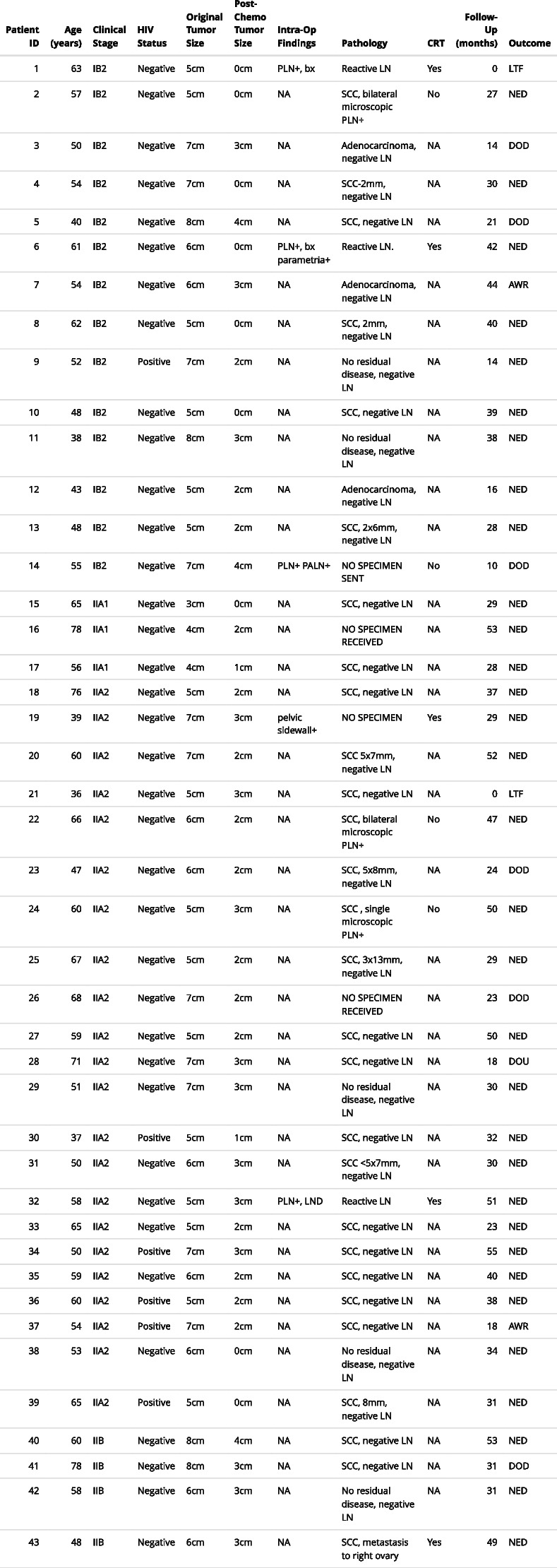
Patient treatment summary and outcomes

*Abbreviations*: *HIV* Human immunodeficiency virus, *PLN* Pelvic lymph nodes, *PALN* Paraaortic lympj nodes, *bx* biopsy, *LND* Lymph node dissection, *SCC* Squamous cell carcinoma, *CRT* Chemoradiation therapy, *LTF* Lost to follow-up, *NED* No evidence of disease, *AWR* Alive with recurrence, *DOD* Died of disease, *DOU* Died of unrelated disease, *NA* Not applicable

Cervical tumor size where reported=depth(mm)x width(mm), when only one dimension reported=depth(mm)

All surgical specimens from completed radical hysterectomy (*n* = 38) were sent for histopathologic analysis. Two specimens were lost. Five of 36 (13.9%) specimens showed no residual cervical disease on final pathology. Three of 36 (4.5%) had pelvic lymph node metastases. One patient had adnexal metastases. Parametrial margins were negative in 32/36 (88.9%) and not reported in 4/36 (11.1%). Vaginal margins were negative in 28/36 (77.8%) and not reported in 8/36 (22.2%). Of the 5 patients who had hysterectomy aborted due to intra-operative diagnosis of metastatic disease, all had clinical disease involvement of the parametrium as well as pelvic and/or paraaortic lymph nodes that was not appreciated on clinical exam pre-operatively. As frozen section diagnosis was not available, decision to abort was make on clinical assessment. Nine of the 43 (20.9%) surgical patients were referred for adjuvant CRT based on high-risk intra-operative (*n* = 5/9) or final pathological (*n* = 4/9) findings. Only 5/9 (55.6%) patients received CRT due to limited access to RT.

The median follow-up time, defined as date of surgery to last contact or death, was 34.4 months (95% confidence interval [CI] 29.5–38.9 months). Two of 43 (4.7%) patients were lost to follow-up after surgery, and 1/43 (2.3%) died of an unrelated cancer. Of the remaining 40 patients, at last follow-up, 32 (80.0%) showed no evidence of recurrence and 8 (20.0%) had a documented recurrence. Seven of the 8 patients with recurrence had received curative surgery alone with no indication for adjuvant CRT (however, in one patient, pathology was lost, and the patient was followed conservatively post-op rather than referred for adjuvant CRT). Of those with recurrence, 5/7 died of their disease at 14, 18, 23, 24 and 31 months, and 2/7 were alive with disease relapse diagnosed at 18 and 44 months. An additional patient with disease recurrence had gross pelvic and para-aortic disease at surgery, hysterectomy was abandoned, and the patient did not receive CRT and died 10 months after surgery. There were no documented recurrences among those who received adjuvant CRT.

**Fig. 2:**
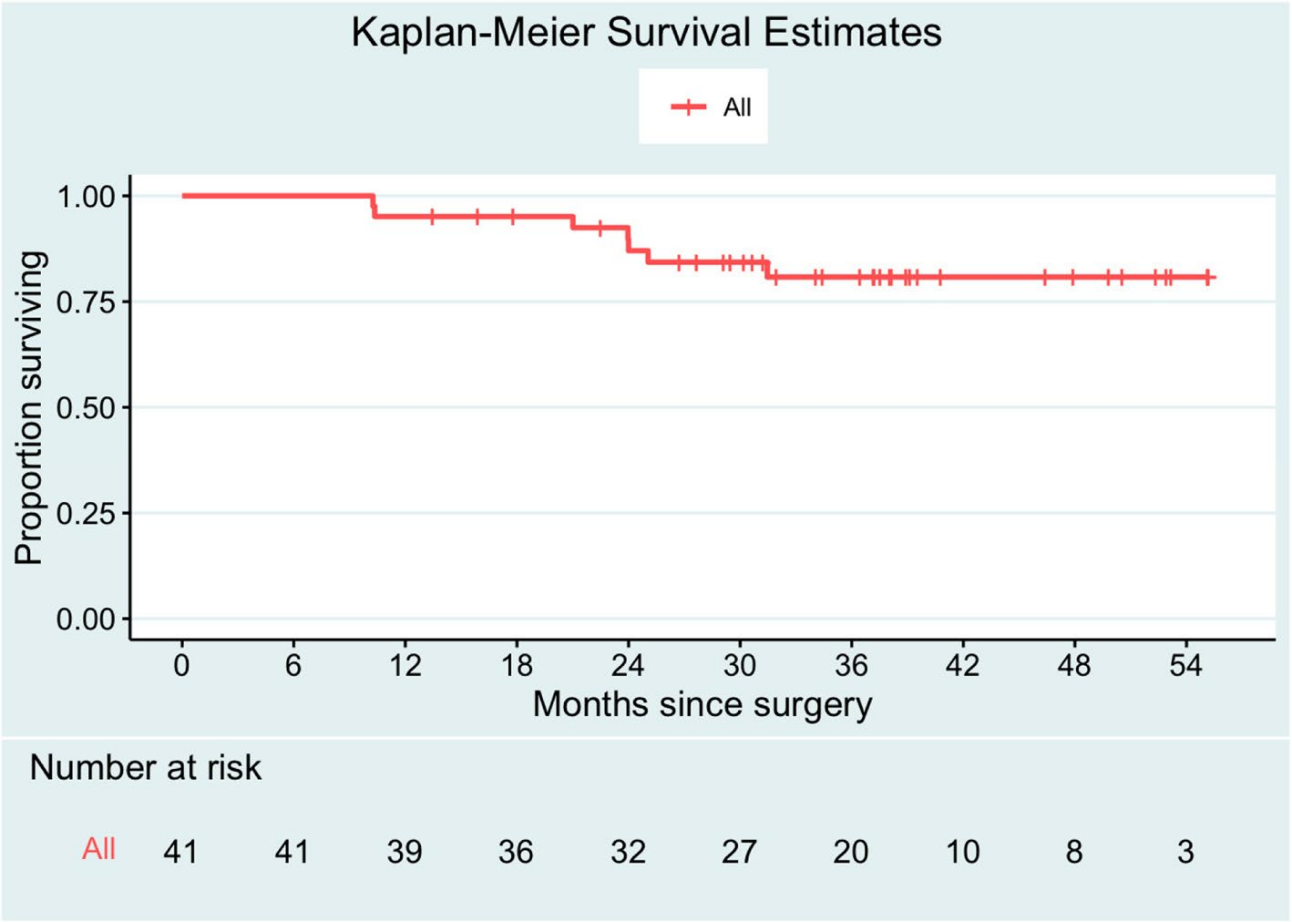
Survival outcomes. Among the cohort, median follow-up time was 34.4 months (95% CI 29.5–38.9 months). Median survival time was not reached. Three-year OS rate was 80.8% (95% CI 68.8–94.9%)

The 3-year OS rate for the 41 patients with follow-up available who underwent NACT-S was 80.8% (95% CI 68.8–94.9%). Median survival time was not reached (Fig. 2). On univariable Cox regression analysis, age, HIV status, stage, preoperative tumor reduction, hemoglobin, and platelets were not found to be associated with worse overall survival (Table 3).

**Table 3:** Factors associated with overall survival: univariable analysis

Characteristic	UVA HR (95% CI)	*p* Value
	*N* = 43 (100%)	
Age (years)	1.01 (0.94–1.08)	0.75
HIV status		
Negative	1 (ref)	–
Positive	–	–
Clinical stage		
IB2	1 (ref)	–
IIA1	–	–
IIA2	0.51 (0.10–2.56)	0.41
IIB	0.80 (0.08–7.74)	0.85
Clinical stage		
I	1 (ref)	–
II	0.53 (0.12–2.37)	0.40
Tumor reduction (cm)	1.14 (0.63–2.08)	0.67
Hemoglobin (g/dL)	0.69 (0.41–1.18)	0.18
Platelets (K)	0.99 (0.98–1.01)	0.43

## Discussion

To date, this study is one of the largest cohort of patients receiving NACT-S for cervical cancer in SSA, within a low-resource setting. Our findings showed that for FIGO 2009 stages IB2-IIB cervical cancer, 75% had a clinical response to NACT making them candidates for radical surgery, and the 3-year OS rate after NACT-S was 80%. The treatment protocol for NACT in the management of cervical cancer in Rwanda was initiated due to limited access to definitive radiation therapy and successfully resulted in expanding access to cervical cancer surgical services. Many women with potentially curative cervical cancer live in settings with limited or significantly restricted access to RT. Therefore, treatment approaches that adapt to the local context are necessary until resources can be improved and capacity increased [15]. The 2021 FIGO cancer report emphasizes that CRT is the preferred mode of treatment for locally advanced cervical cancer, yet treatment options depend on the availability of resources [16].

As of 2020, radiation therapy facilities are unavailable in 25 of 54 African countries [17]. Even in countries with radiation therapy facilities, many patients experience long delays in treatment initiation due to limited access. In Nigeria, for example, the average waiting time for radiation therapy is 175 days [18]. Similar delays have been reported in time from diagnosis to initiation of CRT in Zambia (106 days) [19], Bangladesh (> 180 days) [13], and two studies in Ethiopia (116 and 137 days) where they have also documented disease progression of cervical cancer to higher stages during the waiting period [20, 21]. Zimbabwe has reported treatment interruptions of radiotherapy services due to machine breakdown and financial challenges [22]. At the time of this study, there were no RT services available in Rwanda and patients had to be referred to Kenya for CRT. Due to cost, a very limited number of cervical cancer patients had access to CRT and those that were chosen often had long waiting times, increasing the chance of disease progression, then making them ineligible for treatment once they arrived at the radiation facility. DeBoer et al. found that only 10% of cervical cancer patients in Rwanda initiated CRT within 60 days of diagnosis and the median time to CRT initiation was 126 days [11]. As a result, an alternative management strategy for locally advanced cervical cancer, especially in settings where radiation therapy is unavailable, is to administer NACT with the aim of shrinking the tumor to then allow safe surgical resection [13, 15]. This treatment approach is listed as an option for stages IB2-IIA2 cervical cancer in the National Comprehensive Cancer Network Harmonized Guidelines for SSA [8] and supported by the American Society of Clinical Oncology resource-stratified treatment guidelines [7].

Surgical resection rates after NACT have ranged from 72 to 91% in various studies [14, 23–28]. A review of NACT in cervical cancer reported the outcomes of these studies [6] including Gupta et al. in India comparing NACT-S to CRT in stages IB2-IIB cervical cancer. They reported a resection rate after NACT of 78.5, and 23.1% of patients requiring adjuvant CRT or RT. They concluded that NACT-S cannot be considered a standard of care for locally advanced cervical cancer based on 5-year disease-free survival (DFS) in the NACT-S versus CRT group of 69.3% versus 76.7%, although there was no significant difference in 5-year OS. Additionally, subset analysis showed that 5-year DFS for NACT-S versus CRT was not significantly different for stages IB2 and IIA, only for stage II B[14]. Kenter et al. in a recent randomized, multicenter study of stage IB2-IIB cervical cancer showed no significant difference in 5-year OS in NACT-S versus CRT but 48% of their patients in the NACT-S group received adjuvant RT or CRT [29]. However, they did not report adjuvant therapy with respect to stage, but most of their patients (57%) were stage IIB. It is important to remember that in both studies, patients had direct access to CRT, which is not the situation in most of SSA. In our cohort of women in Rwanda, we focused NACT-S on this early advanced staged group (IB2-IIA), including only a few IIB patients who would not be able to receive CRT due to social circumstances. Like the India study, 75.4% of patients had a response to chemotherapy adequate to allow surgery and ~ 20% of our patients who underwent surgery were referred for RT. Considering the limited radiation services available and the 5-year OS for stages I-II cervical cancer in Sub-Saharan Africa reported at 50.3% [5], the use of NACT-S appears to be a reasonable treatment option in the absence of RT. Even in more advanced stage disease, as most patients in SSA present, this would be an option if no radiation is available. Otherwise, patients are simply left with palliative measures without undergoing any attempt at curative treatment.

As RT facilities are lacking in LMICs, so too are adequately trained gynecologic oncology surgeons. Estimates show that of women diagnosed with early-stage cervical cancer in sub-Saharan Africa, 93% do not have access to safe, timely, and affordable surgery, including radical hysterectomy and fertility-sparing surgery [30]. Although strides are currently being taken to increase training of gynecologic oncologists in LMICs [31], alternative treatment methods are needed in those countries lacking both RT and surgeons trained in oncologic resections. In settings where there are no specialty-trained gynecologic oncologists, general gynecologists have performed cervical cancer surgery with some reported success. In Botswana, in a pilot study of stages IA2-IB1 cervical cancer patients (*n* = 8), NACT followed by simple hysterectomy and pelvic lymph node sampling performed by general gynecologists resulted in a cause-specific survival of 100% over 3.5-year time-period [32]. More studies are needed to define the feasibility of a larger adoption of this approach outside of a tertiary hospital setting. Another adaptive approach to the lack of gynecologic oncology surgical specialists has been described by Hicks et al. as ‘competency-based focused surgical intensification’. This approach entails a rapid transfer of surgical skills training for a general gynecologist to be able to perform radical abdominal hysterectomy and pelvic lymphadenopathy in countries such as Malawi and Democratic Republic of the Congo (DRC), where gynecologic oncologists are few [33].

We acknowledge some key limitations in our study. Patients in our study were assessed preoperatively by clinical staging exam and bedside ultrasound. Radiographs for patients at initial diagnosis to exclude obvious lymph node or distant metastasis would be optimal. This was initially considered for our patients, but when computer tomography (CT) scans were ordered, it significantly delayed treatment and, in many cases, could not be obtained due to financial constraints and patients were lost to follow up. Therefore, we relied on clinical exam only to stage patients and initiate therapy. It is likely that the patients who were found to have gross metastasis intra-operatively would have been upstaged by baseline CT scans and referred for CRT. However, we also consider this a strength, as our results reflect real-world decision making in many LMICs where advanced imaging is often not readily accessible and treatment plans are based on clinical exam and limited radiographs [30]. Our study was also limited by the short follow-up period of our patients and lack of a comparison group receiving CRT. We were unable to report data on NACT tolerability and side effects, as chemotherapy was not administered at our study site, CHUK, but at BCC in the Northern Province of Rwanda.

Additionally, the pathology reports used for decisions about adjuvant treatment in our cohort often had incomplete or missing information, and in some cases missing specimens. Ideally, histopathological prognostic factors after radical hysterectomy, including deep cervical stromal invasion, parametrial infiltration, lymphovascular space involvement, and lesion size, should guide the decision for adjuvant therapy [34]. Of the patients who received NACT and surgery and had a complete surgical resection with no high-risk features documented on final pathology report, 7/40 (17.5%) developed a recurrence, suggesting possible underreporting of high-risk features. Utilizing a more stringent histopathologic criteria for recommendation of adjuvant CRT may have reduced recurrence rate by treating patient at high-risk for recurrence. Pathology services in Rwanda have significantly improved since this study; however, this limitation may have contributed to under-treatment in the adjuvant setting and treatment failures. Lack of adequate pathology reports is certainly experienced by other LMICs as the average number of pathologists per capita in Africa is 1/1,000,000, compared to 1/20,000 seen in the USA and UK [35, 36]. The skills of pathologists specialized in gynecologic oncology is severely restricted in LMIC, resulting in lack of standardized pathology reporting.

## Conclusions

NACT-S is a feasible treatment option for locally advanced cervical cancer in low-resource settings in the absence of RT. Although not currently considered standard of care, it can be an alternative treatment option in countries without radiation facilities if gynecologic oncologist or gynecologists skilled at radical surgery are available. As we continue to push to expand access to cervical cancer treatment throughout Africa, increasing the number of trained oncologic surgeons could provide much needed timely care and reduce the burden on limited radiation services while providing curative surgeries. It is critical for areas with limited resources to share their outcomes of these alternative treatments to determine if they are feasible, safe and have acceptable patient outcomes, when preferred treatment options are unavailable or significantly limited.

## Data Availability

The datasets used and/or analyzed during the current study are available from the corresponding author on reasonable request.

## Abbreviations

LMIC: Low- and middle-income countries
SSA: Sub-Saharan Africa
CRT: Concurrent chemotherapy and radiation therapy
FIGO: International Federation of Gynaecology and Obstetrics
NACT: Neoadjuvant chemotherapy
NACT-S: Neoadjuvant chemotherapy followed by radical surgery
RT: Radiation therapy
UCI: Uganda Cancer Institute
CHUK: University Teaching Hospital of Kigali
BCC: Butaro Cancer Center
DFS: Disease free survival
OS: Overall survival
